# Experiences of residents of the San Ignacio University Hospital in Bogotá DC, Colombia during the COVID-19 pandemic: a qualitative study

**DOI:** 10.47626/1679-4435-2024-1017

**Published:** 2025-01-07

**Authors:** Valentina Lugo-Mesa, Carlos Jose Villota, Maria Paz Andrade, José Antonio Garciandía, Francisco Palencia-Sánchez, Yazmin Cadena-Camargo

**Affiliations:** 1 School of Medicine, Pontificia Universidad Javeriana, Bogotá, Cundinamarca, Colombia; 2 Department of Preventive and Social Medicine, School of Medicine, Pontificia Universidad Javeriana, Bogotá, Cundinamarca, Colombia

**Keywords:** mental health, COVID-19, community health workers, doctor-patient relations, psychological distress, salud mental, COVID-19, cuerpo médico de hospitales, relaciones médico-paciente, agotamiento psicológico

## Abstract

The COVID-19 pandemic has caused a major health crisis around the world. Health professionals have contributed with all their abilities to provide adequate medical care in a scenario of great uncertainty and with many difficulties. The objective of this article is to investigate the experiences narrated by the residents of the San Ignacio University Hospital, in Bogotá DC, Colombia, during the pandemic. A qualitative study was carried out in which 15 in-depth interviews were conducted with residents of different medical specialties, which allowed us to explore the difficulties, challenges, opportunities, and capacities they have faced during the pandemic. Through a thematic analysis, the following categories were identified: “Working with COVID-19”, “Building relationships”, “Learning during a pandemic”, and “How did residents feel?”. We found an increase in workload, limitations in the learning tools, overwhelming feelings and augmented tensions in work and social interactions, all this due to the health system in crisis, the prioritization systems implemented, as well as the increase in the number of deaths despite the effort to preserve life. As a recommendation, we propose a reform to the Colombian residency program, with main emphasis on the academics and a greater recognition of the work they do, as well as providing them with tools that helps them manage their emotions for the maintenance of their well-being and mental health.

## Abstract (es)

La pandemia por COVID-19 ha provocado una importante crisis sanitaria en todo el mundo. Los profesionales de la salud han contribuido con todas sus capacidades para brindar una adecuada atención médica en un panorama de gran incertidumbre y lleno de dificultades. El objetivo del presente artículo es indagar sobre las experiencias narradas por los residentes del Hospital Universitario San Ignacio, de Bogotá DC, Colombia, durante el transcurso de la pandemia y formular conclusiones a partir de las cuales realizamos recomendaciones para mejorar la salud mental de los residentes. Se realizó un estudio cualitativo en el cual se realizaron 15 entrevistas a profundidad a residentes de diferentes especialidades médicas, las cuales nos permitieron explorar las dificultades, retos, oportunidades y capacidades a las que los residentes se han visto enfrentados durante la pandemia. Mediante un análisis temático, se identificaron las siguientes categorías: “Trabajando con la COVID-19”, “Entablando relaciones”, “Aprendizaje durante una pandemia” y “¿Qué sienten los residentes?”. Se encontró un aumento en la carga laboral, limitaciones para el aprendizaje, sentimientos negativos y tensiones en las interacciones sociales laborales y extralaborales, debido al sistema de salud en crisis y a los sistemas de priorización implementados, así como el aumento en el número de casos de fallecimientos a pesar del esfuerzo en preservar su vida. Como recomendación, planteamos la implementación de actividades al programa de residencia colombiano con principal énfasis en la parte académica y mayor reconocimiento a la labor que realizan, así como también brindarles herramientas que les permitan un mejor manejo de sus emociones para el mantenimiento de su bienestar y salud mental.

## INTRODUCTION

Since March 11, 2020, when the pandemic due to the COVID-19 virus was declared by the World Health Organization (WHO), approximately 182 million cases have been reported worldwide as of the date of this study,^1^ a situation that has posed a major challenge to health care services, forcing them to restructure themselves in order to ensure service provision with all the available human and technological resources. This has led health care workers to experience several situations of stress and work overload due to the high number of patients they need to take care of, in addition to the consequences of the use of personal protective equipment (PPE), isolation, uncertainty, and high number of deaths.

As of the writing of this article, more than four million cases and more than a hundred thousand deaths were reported in Colombia, making it one of the 10 leading countries in number of deaths worldwide and the fourth leading country in Latin America, after Brazil, Mexico, and Peru.^1^ This has generated a health crisis in which a considerable proportion of hospitals nationwide declared a state of functional emergency due to the high number of patients and the high occupancy of intensive care units (ICUs), which could reach more than 80% in the Colombian territory in July 2021.^2^

In the Colombian context, residents are medical graduates currently licensed to practice their profession in Colombia and who belong to a medical-surgical specialization program in health institutions certified by the Ministry of Health and Social Protection, where they undertake their theoretical and practical studies.^3^ This position places them in a special situation as students and as health care professionals at the same time, with all the responsibilities of a general physician and of the specialty they are studying. Currently, as first line health care workers, residents also must face all the changes resulting from the pandemic, both in the work and academic contexts, becoming a group of interest in terms of social vulnerability.^4^

As students of a medical specialty, because of the demands of graduate programs, most residents must be dedicated exclusively to the program, but are also required to perform different work activities (shifts, medical procedures, compliance with time schedule, among others) which leads to an overload of in-person academic activities. For this reason, Law 1917 was approved in Colombia in 2018 including monthly remuneration and affiliation to the General Health System and to the General Occupational Risk System.^5^

Health care workers have been confronted with the effects of the pandemic, seeing firsthand the overload of the health care system, the collapse of health care networks, and even contagion with the virus and the possibility of dying serving the cause, since only in Bogotá 260 deaths and a total of 50,405 cases were reported, according to the report by the Colombian National Health Institute as of March 19, 2020.^6^ During the epidemiological peak, health care professionals had to deal with lack of PPE and scarcity of essential medical resources.^7,8^

In different qualitative studies conducted in other countries, the following situations have been reported in health care professionals: concerns due to constant changes in clinical practical guidelines, concerns due to deficiencies in training to deal with COVID-19, shortage of PPE and resources, as well as fear of contagion and of contaminating their relatives.^9,10^

Furthermore, different impacts have been identified on health care professionals’ mental health, such as the onset of mental disorders and feelings of fear, anxiety, and frustration. However, during the pandemic there has also been a greater feeling of solidarity among peers and the use of emotional support groups as measures to cope with stress and the emotional repercussions emerging from the pandemic.^9^

Currently there is a limited number of articles on the life experiences of residents as a special group during the pandemic. Therefore, the aim of the present article is to investigate the work experiences, difficulties, challenges, and opportunities of residents at Hospital Universitario San Ignacio (HUSI), as well as changes and impacts on their mental health when experiencing great emotional stress.

### THEORETICAL FRAMEWORK

#### Burnout syndrome

The concept of burnout was defined by the American social psychologist Christina Maslach, who coined it as a syndrome characterized by emotional exhaustion, depersonalization, and reduced personal accomplishment at work, which usually occurs among individuals whose daily tasks involve interacting with people.^11^ This concept shows that working conditions can have a negative effect on workers’ health and that this effect is produced not only at the physical level, but also have implications in mental health.

With the concurrent pandemic, there has been a significant increase in the prevalence of this syndrome, which has been associated with significant psychological changes in health care workers.^12^ In a study about the mental health of staff working in ICUs during the first wave of COVID-19 in the United Kingdom, it was found that, among 709 participants, 45% reported symptoms of probable depression, post-traumatic stress disorder, and severe anxiety, whereas in a survey-based study with 136 professionals working in an ICU in Milan, Italy, up to 60% met criteria for burnout.^2,13^

Particularly among health care professionals, medical graduate students are the most vulnerable population. This is especially due to their exposure to different situations of suffering and death, in which constant contact with patients and their families demands a higher level of commitment, making of important decisions, and greater responsibility. It has been evidenced that residents present even higher levels of burnout than medical specialists, due to their lack of experience.^4^ Therefore, it is crucial to improve understanding of the effect of different labor stressors on the mental and physical health of health care workers, especially of personnel in training, during this global crisis in the different health systems, and particularly in Colombia.

### WORK RESILIENCE

The term resilience comes from the Latin word *resilio*, which refers to return to a previous state by jumping or rebounding. The first studies on resilience conducted by psychologists such as Anthony in 1974 and Werner and Smith in 1982 focused on the individual characteristics present in childhood and adolescence that allow to respond to stressful situations. This concept was modified by the psychiatrist Michael Rutter, who stated that resilience was also influenced by external aspects, including family, community, and cultural factors. One of the most recent definitions of this word was created by Luthar, for whom resilience, in short, is the ability to positively adapt to adversities.^14^

Resilience has a great impact on the work environment, since it is considered an essential factor to react properly to stress. It is especially used by professionals experiencing high levels of stress, as has been the case of health care workers during the current pandemic. Therefore, resilience has been considered a mental health predictor. A systematic review was conducted in November 2020 to assess the use of interventions promoting resilience and mental health in health care workers during and after epidemics and pandemics. It was concluded that the greatest existing obstacles for the appropriate implementation of strategies to promote resilience in the work context include lack of time, skills, and equipment required to perform an appropriate intervention.^15^

## METHODS

A descriptive exploratory study was conducted to understand the experiences of residents of the HUSI during the COVID-19 pandemic, who were part of the first line of care for this disease, as well as hospital staff in training.

### TARGET POPULATION AND COLLECTION OF INFORMATION

Interviews were conducted with residents of different specialties and with a labor contract with the HUSI. Convenience sampling was used among all the specialties available in the hospital, considering the following inclusion criteria: active employment status with HUSI during the COVID-19 pandemic and willingness to participate in the interviews. The only exclusion criterion was having a terminated employment relationship with the hospital.

Fifteen in-depth interviews were conducted, in which residents were asked about their experiences during the pandemic. Due to the conditions derived from the pandemic, interviews were conducted through the telephone, with previous participants’ informed consent. Each interview lasted approximately 30 minutes, and recordings were deleted, but interview transcriptions were maintained.

Information was collected at the HUSI, a fourth level referral institution located in Bogotá DC, Colombia, from February to March 2021.

### ETHICAL CONSIDERATIONS

At the time of the study’s development, all participants were aged between 25 and 36 years. At the beginning of the interviews, all aspects of the study were clearly explained, and verbally informed consent was obtained from each participant. They were informed that they could withdraw from the interview at any time. Recordings of the interviews were deleted after being transcribed, and these transcriptions were stored. According to the Colombian law no. 8430 of 1993, this study presents minimal risk to participants. This study was approved by the Research Ethics Committee of Pontificia Universidad Javeriana School of Medicine and by the HUSI, through protocol No. FM-CIE-0938-20, of September 18, 2020.

### ANALYSIS

Information was systematized, and the process of codification and categorization resulted in six predetermined codes (stress and work experience, changes in personal and family life, and skills such as resilience) and five emerging codes (teamwork, discrimination, virtuality, solidarity, and academy). Subsequently codes were classified into the following analysis categories: “Working with COVID-19”, “Building relationships”, “Learning during a pandemic”, and “How did residents feel?”.

## RESULTS

After an abductive analysis of the interviews, codes were classified into the following categories: “Working with COVID-19”, “Building relationships”, “Learning during a pandemic”, and “How did residents feel?”.

Findings are presented below.

### WORKING WITH COVID-19

Much tension was created between residents and professors and between residents themselves, initially to define who would manage patients with COVID-19 and who would not, since fear of contagion and uncertainty on the treatment and prognosis of this disease persisted.

*“Of course, since everything has began and all of a sudden, especially at the beginning, there was much tension and, in general, the fear we all felt that we could get contaminated, uncertainty of the unknown, not knowing how to go see the patients, so all this made the environment a little bit more tense in general”* (Sara, emergency medicine resident).

Furthermore, because there was a higher number of patients with respiratory diseases, the use of complete PPE was required, and the care for these patients demanded more time from the resident in terms of data collection, physical examination, completing patient’s epidemiological registry, executing mandatory isolation, formulating the diagnosis, and lastly explaining to patients their situation and the therapeutic plan after establishing a good doctor-patient relation.

*“In the end the situation generated stress and in a certain way was also time-consuming, it was necessary to ask patients whether they had contact or not, it was necessary to examine them well, because obviously it often depends on us whether the patients stayed in hospital or went home and, um, doing things like completing the epidemiological registry and such…”* (Sandra, geriatrics resident).

Other residents of specialties that did not need to treat respiratory disease reported a change in their role, because they had to perform functions different from that of their specialty, due to the reduced number of patients treated and therefore less exposure to diseases that concern their specialty. An example is the case of Sebastian, a plastic surgeon resident who reported that:

*“Regarding the work aspect, the number of patients decreased very much, especially in my specialty, which is plastic surgery, our impact has been more related to a decrease in the amount of work and not to increased workload”.*

Therefore, residents were affected in their graduate training, because of the reduced variability in clinical scenarios, which can also contribute to occupational stress.

Uncertainty in the management of patients with COVID-19 was resolved with the clinical practical guidelines established by the Ministry of Health and by the hospital. Although these guidelines had constant changes and were a matter of debate and discrepancies on many occasions, they were an anchoring point for uniform decision-making, especially for inexperienced residents.

*“I believe that, when one is governed by a guideline, by evidence, by experts’ support, it works much better, both in decision-making about patients and use of personal protective equipment and hand washing, I believe that guidelines kept us afloat because they allowed us to speak the same language, make the same decisions, and being able to resolve everything as a group.”* (Pedro, emergency medicine resident)

Residents reported that it is important to individualize and adapt guidelines for each patient. This shows where lies the importance of the doctor, the person wearing the coat, who is not only the one who applies a series of protocols, although COVID-19 is a disease that manifests similarly in most cases. They should put their analysis into context and create a rapport with each patient to provide them with comprehensive and humane management.

*“Obviously clinical practice guidelines, because they are exactly this, guidelines that lack information on certain things related to humanity, right? For example, guidelines will never tell you that patients with worsening respiratory status should be provided with comfort measures to remain comfortable if they don’t have a better prognosis so that they could have, let’s say, a peaceful death. It is exactly what we, doctors, do, also following them up and alleviate some symptoms in the let’s say impending death.”* (Juan, family medicine resident)

For instance, residents who treated senior patients had a direct confrontation with guidelines indicating that patients older than 80 years of age could not be prioritized. However, residents themselves needed to provide many arguments to protect this population, despite being against protocols, showing their perseverance in defending the most vulnerable and providing them with the opportunity to live.

*“There was time when controversy emerged about an institutional guideline recommending that those older than 80 should not be admitted to the intensive unit care, but we obviously were always against this recommendation and continued to refer our patients to this unit and tried that they received the care they need…I understand that a prioritization list exists, but it should consider all the variables.”* (Sandra, geriatrics resident)

Another factor that had a great impact on everyday changes was use of PPE. Many residents mentioned the importance it gained in their lives and the implications it had in their social interactions, because it hampered verbal and non-verbal communication, thus limiting the establishment of an empathetic relationship, an essential aspect in patient management.

*“Learning to take some distancing measures when seeing people, and, for instance, hand washing, which was not done so often before, having to wear a mask all the time, not being able to kiss or hug relatives and friends, to me this seemed to have a great impact because in the end it is what reduces the chances of contamination.”* (Juan, family medicine resident)

Considering the use of protective equipment and hygiene measures implemented to prevent the spreading of the virus, residents learned about the importance of selfcare on the well-being of their patients, which was not previously considered important when treating them. *“I surely do emphasize that it has taught us to care for us as health care professionals who are exposed to many diseases, not only respiratory ones but also those that may be contagious”* (Sergio, internal medicine resident).

### BUILDING RELATIONSHIPS

Amid these difficulties, residents found their family frustrated and confused, and residents themselves felt the same, because there was little information about the disease and there was little to be done. Furthermore, isolation and the high number of deaths generate a very tense environment, thus worsening communication between residents and their families. Poor communication resulted in a conflictive doctor-patient relation, and the same occurred with their relatives, which perpetuated negative feelings. This emotional baggage eventually affected residents’ mental health.

*“Telling the patients and also their family is very complex and hard, explaining the situation to patients… it is one of the most complicated things that one can experience, because nobody has ever experienced this before, and explaining that presently there are no resources available to provide proper care, it remains hard for them to understand, this also generates some anxiety in their families.”* (Sergio, internal medicine resident)

Residents described how their interpersonal relationships were also affected, since many of them had to leave their families, move from their homes, or cut any communication with them, in order to protect their relatives from possible contamination.

*“Thus, limiting the part of sharing with our family, most of us tried to isolate ourselves and avoid contact with our loved ones”* (Sara, emergency medicine resident).

Nevertheless, distancing from family perpetuated residents’ feelings of loneliness and sadness, since family is their main support network. However, many of them preferred not to share this emotional burden with their relatives in order to avoid transmitting the feeling of anxiety to them. Furthermore, in relation to residents’ personal life, there was a distancing from their friends, their peers, and the rest of society, which perpetuated the situation.

*“The issue of having to be distant from one’s family, because my parents live in another town and it’s not easy to go see them, be with them, this affected my mood, one needs the emotional support from their family in a household, it was something that I had to learn to cope with.”* (Pedro, emergency medicine resident)

Interpersonal relationships deteriorated, since residents could not share face-to-face time with friends and colleagues. Many of them were forced to resort to new ways of communication using technology to establish remote contact and not lose contact.

*“We always had breakfast after the clinical case on Fridays, we had inter-weekly meetings, and we tried to hold these meetings virtually, but it obviously was not the same. Because there was a time that we didn’t see each other… So I believe that in this sense there was indeed a deterioration in this unit compared with what it was before.”* (Juan, family medicine resident)

Residents’ social life had significant changes as well, e.g., in terms of their everyday social activities. Social isolation and total immersion into the hospital environment made residents reflect about all they were forced to quit doing because of the pandemic. Activities that seemed common before had to be completely abandoned, but there was hope that someday life would return to normal.

*“Being able to go to the movies, which one did not enjoy so much before and seemed like something that could happen any day, and if one looks at it today, I believe that most of the people haven’t been to the movies for more than a year, and haven’t meet their friends, which was something silly before, but nowadays, one says that when they can go back to the movies and all those things, it should be great.”* (Valentina, radiology resident)

All these changes not only had an impact on residents’ emotional state but there were also secondary changes at the physical level that ultimately affected their mental health. *“I do feel that these sudden changes made my mood change and in fact I began to eat more and gained weight”* (Sandra, geriatric resident).

A relevant topic that we considered appropriate to investigate was whether residents had suffered discrimination because they were health care workers. None of them reported acts of discrimination inside the hospital, and only one of them mentioned suffering discrimination by close persons. *“…with my friends I did have problems, I’ve recently found out that there was a time when they did not want to go out with me due to my exposure, so I found out that they had made plans like 1 month later”* (Sergio, internal medicine resident).

### LEARNING DURING A PANDEMIC

In relation to the academic context, the most important change was the transition from face-to-face to virtual learning, which allowed for the implementation of new tools to continue with residents’ training process and protect their health and prevent contamination at the same time. This brought both positive and negative aspects. Among the positive ones, it is worth highlighting that virtual learning made information and education universally available to everybody, despite the circumstances, and facilitates communication between colleagues so as to improve patient care. *“Virtual learning has been elementary for our training. In other words, without virtual learning this would have totally collapsed, and we could not have continued in the residency program…”* (Camilo, internal medicine resident).

Regarding negative aspects, many residents reported that virtual education makes it difficult to focus, prevents appropriate interaction with professors, and does not allow for examining and observing the patient inside the hospital environment for comprehensive learning. *“Because it is obviously much harder, and I believe it happens to everyone, for one to follow a 2-hour lesson, sometimes longer, virtually”* (Juan, family medicine resident).

### WHAT DID RESIDENTS FEEL?

Like everyone else, residents report that the pandemic was an unexpected event and that they felt much uncertainty, because they did not know how the virus was transmitted and how to diagnose and manage it. Furthermore, they shared feelings of anxiety and concern, since they were forced to deal with the disease with no other tool than their willingness to help the patient.

*“Of course, since everything began and all of a sudden, especially at the beginning, there was much tension and, in general, the fear we all felt that we could get contaminated, uncertainty of the unknown, not knowing how to go see the patients, so all this made the environment a little bit more tense in general.”* (Sergio, internal medicine resident)

Residents report that the most difficult time was the beginning of the pandemic, with all the fear of having to deal with so many difficulties and the lack of time to realize the full extent of what was happening and how it affected their mental health, a situation resulting from the fact that residents only had time to work and study, since the country, the health care system, and patients’ life depended on them. They should put their feeling and their own health aside to watch and care for the health of the other Colombians.

*“…It was frustrating for everybody, it was a time when we had to do, do, do, and when we stopped for sometimes a second to breathe, take a breath, and continue, no matter how sad we felt, we could not give up at that time, we had to go on.”* (Camila, internal medicine resident)

Another difficulty was increased workload, together with the monotony of the disease residents were treating, in addition to the high number of patients and realizing that, despite their efforts, outcomes were not satisfactory. *“At the beginning, one day was dedicated only to seeing respiratory patients, it was actually very tiring, one felt very overwhelmed”* (Camila, internal medicine resident).

Residents also cope with an emotional burden that they had never experienced before, because most patients had a dismal prognosis, despite all efforts made by residents for these patients to overcome their illness. It was hard for the residents to realize that all work was in vain most of the time. *“There were young people who eventually died from the disease, so I think that it was very overwhelming and very tiring, mental fatigue from thinking in many situations, I believe that one ended up exhausted”* (Sergio, emergency resident).

Decision-making was also a difficult aspect. Because of shortage of resources, it was necessary to organize a prioritization system that was unfair and primitive, but it was the only way to distribute resources. Together with the heavy workload, this led to feelings of desensitization and dehumanization, which residents used as a defense mechanism against the administrative difficulties that arose and that ultimately determined medical conduct.

*“I believe that sometimes there were so many patients, so many emergency patients, one eventually does not see them as 100% human, one rather wants to detach from this suffering in order to not be affect by the patients, and one tries to somewhat dehumanize care, so to speak, but it is impossible…”* (Camila, internal medicine resident)

A prioritization system must be applied by specialists and their residents, which was difficult for them, because it often meant to limit the therapeutic effort for patients who wished to continue living. Nobody had prepared them to give this type of bad news, they had to arm themselves with courage, seek help from their professor, and explain the difficult situation that eventually affects patients’ lives.

*“So it becomes a dilemma [prioritization], because you think: if it was your uncle or your granddad, would you prioritize them and involve personal matters? It should be used as the last resource, because one never wants to make one life more important than the other, but if we, as doctors, try to seek for the best opportunities to preserve life, unfortunately we have to use scales, but because there is no other resource.”* (Sandra, geriatric resident)

*“…Decision-making at critical times when we ran out of resources and had to prioritize, um, it was tough trying to explain to patients and their families how this prioritization is done and, in very harsh words, how a group of people decide whether someone lives or dies in such an arbitrary way only because there are no resources. So, emotionally speaking, it was the most impacting aspect of the pandemic to me.”* (Sergio, internal medicine resident)

When the peak decreased, residents had some rest and it was when they became aware of the impact of the pandemic on them. They even make explicit reference to onset of burnout, due to all the aspects previously mentioned, and to the effects of these changes on the way they addressed patients. *“…in relation to care and emotional burden, and everything that residency implies, one undergoes like burnout scales and this type of things, and I feel that I’ve lost humanity towards patients…”* (Juan, emergency resident).

Seeing so many people, in whom residents saw their own relatives reflected, dying in such a short time led them to value life more highly. They also understood the importance of cherishing and sharing even the few moments of physical or virtual contact. *“This makes one more humane, makes one cherish their own life and that of their relatives more, and actually turns the time with one’s family and friends much more special”* (Sara, emergency medicine resident).

Some residents also said that the pandemic helped them develop a greater connection with their patients, empathize with them, and provide them not only with medical but also with emotional support.

*“One becomes a little more humane than before, because seeing this difficult situation… A situation in which there is a pandemic affecting many families, many people could not see their relatives the way they wanted… Possibly accompanying them at their deathbed…”* (Camila, internal medicine resident).

Likewise, some residents mentioned the importance of physical activity and leisure activities such as drawing and painting and of family and friends’ support to cope with academic, work, and emotional burden, as a tool to maintain their happiness, especially during pandemic peaks.

*“I like cycling very much, but I quit doing it because there was no bicycle lane or anything, but indoors I like drawing and I do it occasionally, I became more engaged in some of these activities during the pandemic… I decided to start doing physical activity, and it has helped me a lot… it helps improve my mood and makes me feel more comfortable.”* (Camila, internal medicine resident)

Finally, it is worth mentioning that, according to residents, socialization of experiences, feelings, and emotions through empathetic and supportive communication was a key factor for resilience against this situation among residents and physicians, including the establishment of Balint groups.

*“These are sessions organized by a psychiatrist from the hospital in which some very hard experiences are told. This is a frequent practice in services like gynecology or surgery, I believe it has never been done in internal medicine, but with the pandemic it became more common. I have never participated, but some colleagues told me that sharing this type of experiences with other people that had similar experiences helps them a lot.”* (Pedro, emergency medicine resident)

## DISCUSSION

The present study made it possible to highlight the experiences and challenges encountered by residents during the pandemic from their perspective. As we all know, COVID-19 have challenged the Colombian health system, which involved long working hours, monotony due to the type of patients, frustration in the management of the lack of resources and the tiresome use PPE and the limitations it caused in the establishment of the physician-patient relation.

Likewise, a great emotional impact was generated on residents, caused by multiple reasons, such as anxiety and uncertainty on how to manage COVID-19 patients, belonging to the patient prioritization committee for resource distribution, and poor prognosis, especially of older adults, who die despite all effort and dedication. A similar study conducted in the United States with surgical residents reported that the pandemic affected learning possibilities because it limited the number of educational scenarios and generated stress and anxiety related to PPE shortage; conversely, the pandemic brought other benefits, such as virtual learning and increased self-confidence in adverse situations.^16^

In a study conducted at King’s College London with 705 participants, including health care workers from different areas, such as physicians and nurses who worked in critical care units during the COVID-19 pandemic, it was found that 45% reported symptoms of probable anxiety and posttraumatic stress disorder.^2^ Likewise, our study observed that the pandemic lead residents to present characteristics compatibles with burnout syndrome, since they report feelings of dehumanization, depersonalization, anxiety, emotional exhaustion, and frustration about the non-beneficial outcomes, especially during pandemic peaks; however, residents manifested their commitment to serve and provide the best care to their patients in their different medical areas.

Another change that residents had to face, especially at the beginning of the pandemic, was fear of contaminating their family. This meant that many of them stopped having physical contact with their family and friends; some of them even moved from home to continue practicing their profession, due to fear of contaminating their family, showing residents’ commitment in taking care of their health and that of their family and friends, so as to prevent the transmission of COVID-19.

However, isolation increased the development of negative feelings and led to the deterioration of residents’ interpersonal relationships, since many of them expressed how essential family support and social contact are. Regarding their personal life, dramatic changes were observed, because residents stopped performing activities that allowed them to de-stress and forget about their obligations at the hospital for a while; furthermore, it was found that the use of PPE changed residents’ way of interacting with their colleagues, professors, and relatives.

During this pandemic, virtual learning became one of the pillars of residents’ academic training. Their lessons and other activities at the hospital became mostly virtual. This tool helped residents to cope with workload; however, it presents some difficulties, such as the fact that it prevents proper interrelationships with professors. However, we believe that virtual learning has benefited residents’ training process.

Health care workers faced the challenge of stigma and discrimination, often in their workplace and its surroundings. These facts occurred in countries such as the United States and India, where health care workers were beaten and threatened to leave their home; moreover, health care workers who did not treat patients with COVID-19 expressed disgust by sometimes refusing to talk to or eat in the same cafeteria than those treating these patients.^3^ Therefore, we asked to our study population whether they had suffered discrimination because they were health care workers and, although one participant reported he had, most residents did not feel discriminated.

A study conducted in Lagos, Nigeria, which interviewed 10 physicians and 5 nurses, found that they underwent heavy work and emotional burden, especially during pandemic peaks. Furthermore, this study evidenced shortage of PPE and financial resources to the health staff; however, despite difficulties, investigators documented residents’ sense of responsibility and resilience.^9^ Residents’ adaptative capacity to face the critical health situation in Colombia was successful through support groups, where residents could share their experiences with other residents, as well as through physical exercise, leisure activities, and family support. In view of these findings, it is possible to state that, despite the current health situation, residents managed to cope with this great health challenge thanks to the support of university, their residency colleagues, physicians, in addition to family and social support.^4^

One limitation to consider in this study was time, since some residents had little time available for the interview; therefore, they could not spend much time answering our questions, which, in some cases, did not allow for the execution of an in-depth interview. Another factor to consider is that interviews were conducted virtually, a situation that made them possible, but did not promote the empathy and trust required to address this very sensitive issue, not enabling the possibility of non-verbal communication, which is important for a study of this type.^17^

## CONCLUSIONS

Our qualitative study contributed to the investigation on the management of a high number of complex patients with an infection caused by an unknown virus.

The new distance learning modality limited access to patients and personal interaction with professors; however, it allowed students to continue their training.

In many cases there was a deterioration in the physician-patient relationship, due to use of PPE and preventive isolation, although it was possible to be close to loved ones using technology.

Furthermore, we understood residents’ perceptions on the COVID-19 pandemic, both positive ones, since it brought a sense of humanity to residents, and negative ones, such as loneliness, sadness, and fear.

We found that residents gained some skills, especially resilience as the ability to overcome adversities through the implementation of multiple tolls that allowed them to improve their mental well-being.

In light of the foregoing, some recommendations can be made for residence programs, such as: encouraging leisure activities in residents’ spare time and promoting group activities with coworkers and teamwork. Moreover, it is important to foster the use of Balint groups and communication training in the physician-patient relation, comply with the schedule set for virtual sessions, and take moments of rest.

In the future, further studies should be performed to evaluate residents’ mental health in the post-pandemic period, in order to assess the long-term effects of the pandemic on mental health.
